# Draft genome sequence of *Enterobacter* sp. I4, an endophytic bacterium isolated from *Aloe barbadensis* Miller

**DOI:** 10.1128/mra.01514-25

**Published:** 2026-03-13

**Authors:** Mpho Mamphoka Nchabeleng, Abraham Goodness Ogofure, Ezekiel Green

**Affiliations:** 1 Department of Biotechnology and Food Technology, University of Johannesburg, Doornfontein Campus 71989, Johannesburg, South Africa; 2 Microbial Pathogenicity and Molecular Epidemiology Research Group (MPMERG), Department of Biotechnology and Food Technology, University of Johannesburg, Doornfontein Campus 71989, Johannesburg, South Africa; Fluxus Inc., Sunnyvale, California, USA

**Keywords:** *Enterobacter *species, endophytes, *Aloe vera*, secondary metabolites, antibacterial compounds

## Abstract

We report the draft genome sequence of *Enterobacter* sp. strain I4, an endophytic bacterium isolated from healthy *Aloe barbadensis* Miller leaves and stems. The 4.7-Mb genome (56.5% GC) comprises three contigs and encodes 4,339 predicted protein-coding genes, providing a genomic resource for investigating endophyte–host interactions and secondary metabolism.

## ANNOUNCEMENT

Endophytes are microbes that colonize internal plant tissues without causing disease and play important roles in plant health, stress adaptation, and metabolic versatility (1–5). Endophyte-derived metabolites offer sustainable alternatives to direct plant harvesting and constitute an underexplored source of antibacterial compounds for drug discovery amidst rising antimicrobial resistance (1, 2, 5–7). We present the draft genome sequence of an endophyte, Enterobacter sp. I4, from *Aloe vera* and its ecology and metabolic potential.

*A. barbadensis* was harvested from Apel village (Limpopo, South Africa 24° 24′ 0″ S, 29° 44′ 0″ E) in March 2021 and was transported on ice to the laboratory in a sterile polyethylene bag. Endophytes were isolated post-sterilization from PBS-homogenized gel-portion (0.1 mL) of cut leaves and stems, following thorough washing, treatment with 5% Tween 20, surface sterilization with 70% ethanol, and 1.5% sodium hypochlorite, with five rinses (2, 3). Endophytes were cultured on nutrient agar at 30°C for 5 days. Colonies were purified and processed for downstream extraction of high-molecular-weight genomic DNA from 24-h nutrient broth culture using the Zymo Research Fungal/Bacterial DNA MiniPrep Kit (USA). DNA concentration and purity were assessed using the NanoDrop ND-2000 spectrophotometer (Thermo Fisher Scientific, USA). Whole-genome sequencing was performed using PacBio SMRT sequencing on the Revio system (Pacific Biosciences) by Inqaba Biotechnical. SMRTbell libraries were prepared by the service provider using the PacBio SMRTbell Express Template Prep Kit 2.0 following the manufacturer’s protocol. High-molecular-weight genomic DNA was mechanically sheared to a ~15-kb target insert size, and fragment size selection was performed during library preparation using AMPure PB bead purification. HiFi circular consensus sequence (CCS) reads were generated using the SMRT Link application following standard PacBio protocols. Adapter sequences were automatically identified and removed during subread processing prior to CCS read generation in the SMRT Link (v10.1) pipeline. The distribution of HiFi read lengths and read quality scores was assessed using the SMRT Link run summary and CCS quality reports generated with default parameters. A total of 9,614 HiFi reads comprising 65.9 Mb of sequence data were generated. *De novo* genome assembly was performed using the GS *De Novo* Assembler (DEC-2021). The assembled genome was polished using the Arrow algorithm (SMRT Link V10.1) to improve consensus accuracy. Genome annotation was carried out using the NCBI Prokaryotic Genome Annotation Pipeline (PGAP, v6.7). All analyses were performed using default parameters unless otherwise stated, and assembly quality was evaluated by CheckM v1.2.2. The average nucleotide identity (ANI) was calculated using FastANI against representative *Enterobacter* genomes retrieved from GenBank, and the highest similarity was 91.44% for *Enterobacter hormaechei* (GCA_019048245.1)*,* while lower similarity of ≤88.94% was observed for *E. bugandensis* (GCA_900324475.1), *E. mori* (GCA_022014715.1), *E. cloacae* (GCA_019047105.1), and *E. cancerogenus* (GCA_019047785.1). Because ANI values were below the 95%–96% species delineation threshold, the isolate was designated as *Enterobacter* sp. closely related to *E. hormaechei*. Secondary metabolite biosynthetic gene clusters (Table 1) were predicted using antiSMASH v8.0 and compared against the MIBiG database (8).

**TABLE 1:** Genome assembly and annotation statistics for *Enterobacter* sp. strain I4

Parameter	Value
Source (date)	Leaves and stems (2021)
Genome size (bp)	4,718,120
GC content (%)	56.5
Number of contigs (with lengths in bp)	3 (2,598,250, 1,434,460, and 685,410)
Protein coding genes (rRNA genes)	4,339 (22)
Scaffold N50 (bp)	2,598,250
Genome completeness (%)	99.43
Genome contamination (%)	0.50
Genome coverage	~25×
GenBank predicted genes (tRNA gene)	4,501 (78)
antiSMASH (total BGCs)	6
Major classes	NRPS-like siderophore, RRE-containing RiPP, terpene precursor, arylpolyene, azole-containing RiPP, and NRP-metallophore (NRPS)

This draft genome assembly is suitable for comparative genomics, phylogenetic analysis, and exploration of endophyte-associated functional traits. However, as the assembly remains at the contig level, resolution of complex genomic regions and complete plasmid reconstruction may be limited. Future work should include plasmid characterization and circularization where possible.

## Data Availability

The whole-genome shotgun project for *Enterobacter* sp. strain I4 has been deposited in GenBank under BioProject accession number PRJNA816151. The draft genome assembly is available under accession number GCA_022669095.1, corresponding to BioSample SAMN26660210. Raw PacBio sequencing reads have been deposited in the Sequence Read Archive under accession numbers SRS27025772 and SRR35986575.
